# Supplementary material for: Green Synthesis of Iridium Nanoparticles from Winery Waste and Their Catalytic Effectiveness in Water Decontamination
Source: Materials (Basel). 2023 Mar 2;16(5):2060. doi: 10.3390/ma16052060 (PMC10004582; doi:10.3390/ma16052060)
Supplement: Supplementary file 1 [file materials-16-02060-s001.zip › materials-2200935-supplementary.pdf]

Supplementary Materials

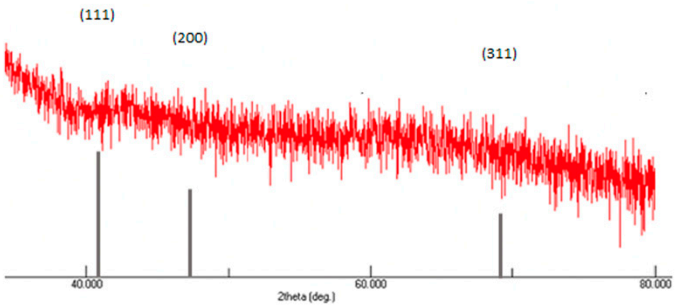

Figure S1. XRD spectrum of Ir-NP2.

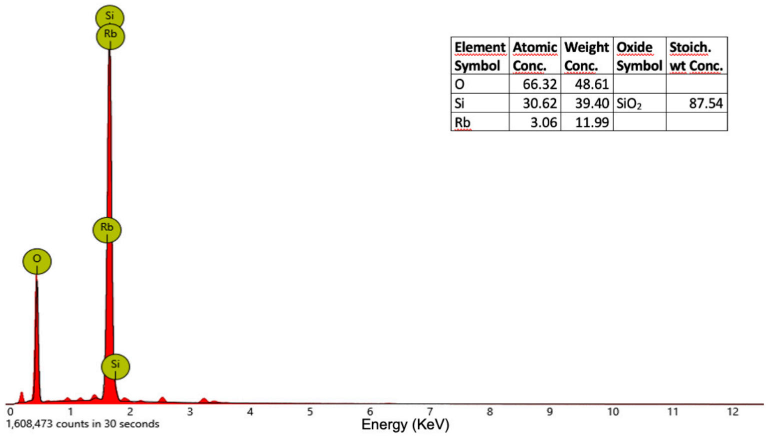

Element Symbol	Atomic Conc.	Weight Conc.	Oxide Symbol	Stoich. wt Conc.
O	66.32	48.61		
Si	30.62	39.40	SiO_2	87.54
Rb	3.06	11.99		

Figure S2. EDX spectrum and elements concentration of wafer substrate.

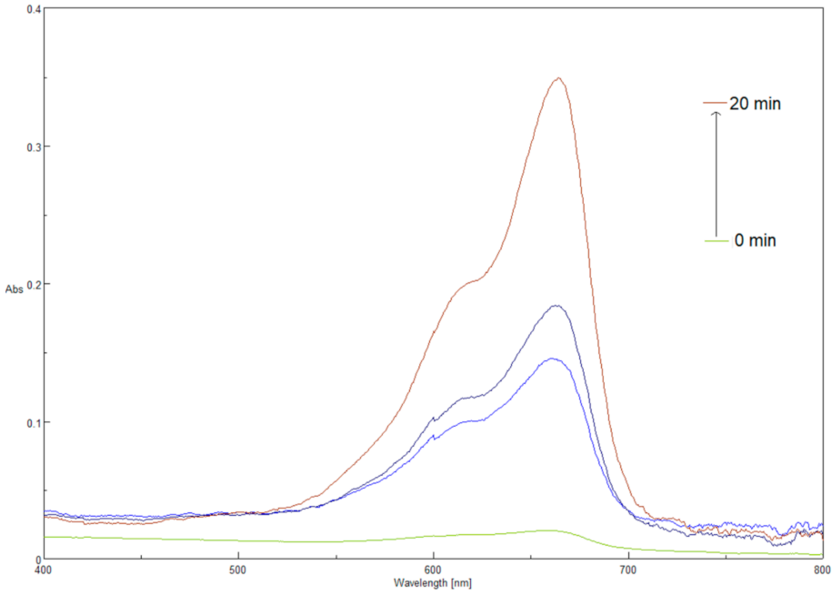

Figure S3. UV-visible spectra registered at different intervals of time of the reaction mixture after catalytic reduction of MB by $NaBH_4$ in the presence of Ir-NP_2 (0 min) and left at the air for 20 min.
